# MeSH Now: automatic MeSH indexing at PubMed scale via learning to rank

**DOI:** 10.1186/s13326-017-0123-3

**Published:** 2017-04-17

**Authors:** Yuqing Mao, Zhiyong Lu

**Affiliations:** 1 Nanjing University of Chinese Medicine, 138 Xianlin Avenue, Nanjing, Jiangsu 210023 China; 2 National Center for Biotechnology Information (NCBI), 8600 Rockville Pike, Bethesda, MD 20894 USA

## Abstract

**Background:**

MeSH indexing is the task of assigning relevant MeSH terms based on a manual reading of scholarly publications by human indexers. The task is highly important for improving literature retrieval and many other scientific investigations in biomedical research. Unfortunately, given its manual nature, the process of MeSH indexing is both time-consuming (new articles are not immediately indexed until 2 or 3 months later) and costly (approximately ten dollars per article). In response, automatic indexing by computers has been previously proposed and attempted but remains challenging. In order to advance the state of the art in automatic MeSH indexing, a community-wide shared task called BioASQ was recently organized.

**Methods:**

We propose MeSH Now, an integrated approach that first uses multiple strategies to generate a combined list of candidate MeSH terms for a target article. Through a novel learning-to-rank framework, MeSH Now then ranks the list of candidate terms based on their relevance to the target article. Finally, MeSH Now selects the highest-ranked MeSH terms via a post-processing module.

**Results:**

We assessed MeSH Now on two separate benchmarking datasets using traditional precision, recall and F_1_-score metrics. In both evaluations, MeSH Now consistently achieved over 0.60 in F-score, ranging from 0.610 to 0.612. Furthermore, additional experiments show that MeSH Now can be optimized by parallel computing in order to process MEDLINE documents on a large scale.

**Conclusions:**

We conclude that MeSH Now is a robust approach with state-of-the-art performance for automatic MeSH indexing and that MeSH Now is capable of processing PubMed scale documents within a reasonable time frame. Availability: http://www.ncbi.nlm.nih.gov/CBBresearch/Lu/Demo/MeSHNow/.

## Background

The rapid growth of scholar publications in biomedicine makes the search of relevant information in literature increasingly more difficult, even for specialists [1, 2]. To date, PubMed—the U.S. National Library of Medicine (NLM) premier bibliographic database—contains over 24 million articles from over 5,600 biomedical journals with more than a million records added each year. To facilitate searching these articles in PubMed, a controlled vocabulary called Medical Subject Headings (MeSH)^1^ was created and updated annually by the NLM since 1960s. Currently, MeSH 2015 consists of over 27,000 terms representing a wide spectrum of key biomedical concepts (e.g. Humans, Parkinson Disease) in a hierarchical structure. MeSH terms are primarily used to index articles in PubMed for improving literature retrieval: The practice of manually assigning relevant MeSH terms to new publications in PubMed by the NLM human indexers is known as MeSH indexing [3]. Assigned MeSH terms can then be used implicitly (e.g., automatic query expansion using MeSH) or explicitly in PubMed searches [4]. Compared with the commonly used keyword-based PubMed searches, MeSH indexing allows for semantic searching (using the relationship between the subject headings) and searching against concepts not necessarily present in the PubMed abstract.

**Fig. 1:**
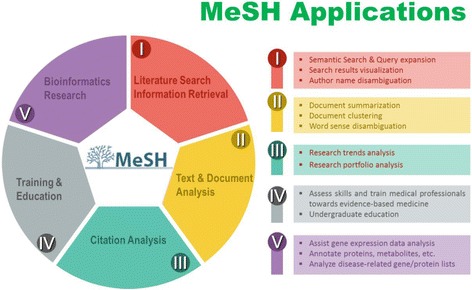
Applications of MeSH

In addition to its use in PubMed, MeSH indexing results have also been used creatively in many other scientific investigation areas, including information retrieval, text mining, citation analysis, education, and traditional bioinformatics research (see Fig. 1). When applied to information retrieval, MeSH and its indexing results have been used to build “tag clouds” for improving the visualization of search results [5, 6] and to help distinguish between publication authors with identical names [7, 8]. Another major use of MeSH indexing is in biomedical text mining, where it has been applied to problems such as document summarization [9], document clustering [10], and word sense disambiguation [11]. MeSH indexing also serves several key roles in citation analysis, from identifying emerging research trends [12, 13] to measuring similar journals [14] and characterizing research profiles for an individual researcher, institute or journal [15]. In the era of evidence-based practice, MeSH becomes increasingly important in assessing and training the literature search skills of healthcare professionals [16, 17], as well as in assisting undergraduate education in biological sciences [18]. Finally, much bioinformatics research, such as gene expression data analysis [19, 20], greatly benefits from MeSH indexing [21–25].

Like many manual annotation projects [26–30], MeSH indexing is a labour-intensive process. As shown in [3, 31], it can take an average of 2 to 3 months for an article to be manually indexed with relevant MeSH terms after it first enters PubMed. In response, many automated systems for assisting MeSH indexing have been previously proposed. In general, most existing methods are based on the following techniques: i) pattern matching, ii) text classification, iii) k-Nearest Neighbours, iv) learning-to-rank, or v) combination of multiple techniques. Pattern-matching methods [32] search for exact or approximate matches of MeSH terms in free text. Automatic MeSH indexing can also be regarded as a multi-class text classification problem where each MeSH term represents a distinct class label. Thus many multi-label text classification methods have been proposed, such as neural networks [33], Support Vector Machines (SVM) [34, 35], Inductive Logic Programming [36], naïve Bayes with optimal training set [37], Stochastic Gradient Descent [38], and meta-learning [39]. While the pattern matching and text classification methods use only the information in the MeSH thesaurus and document itself, the k-Nearest Neighbours (k-NN) approach takes advantage of the manual annotations of documents similar to the target document, e.g. [40, 41]. Additional information, such as citations, can also be utilized for automatic MeSH indexing. For example, Delbecque and Zweigenbaum [42] investigated computing neighbour documents based on the cited articles and cited authors. More recently, Huang et al. [3] reported a novel approach based on learning-to-rank algorithms [43]. This approach has been shown to be highly successful in the recent BioASQ^2^ challenge evaluations [44–46] and has also been adopted by many others [47, 48]. Finally, many methods attempt to combine results of different approaches [49, 50]. For instance, the current production system in MeSH indexing at the NLM is called Medical Text Indexer (MTI), which is a hybrid system that combines both pattern matching and k-NN results [51] via manually-developed rules and continues to be improved over the years [52, 53]. The proposed method in this work is also a hybrid system but unlike MTI, which only uses machine learning to predict a small set of MeSH terms, it combines individual results and ranks the entire set of recommendations through machine learning instead of heuristic rules.

Despite these efforts, automatic MeSH indexing remains a challenging task: the current state-of-the-art performance remains at about 0.6 in F-measure [54]. Several factors contribute to this performance bottleneck: First, since each PubMed article can be assigned with multiple MeSH terms, i.e. class labels, the task of automatic MeSH indexing can be seen as a multi-class classification problem. In this regard, the size of the MeSH vocabulary makes automatic classification challenging: 2014 MeSH includes more than 27,000 main subject headings and they are not equally used in indexing [31]. Second, MeSH indexing is a highly complex cognitive task. It has been reported that the consistency between human indexers is only 48.2% for main heading assignment [55]. Lastly, both the MeSH vocabulary and indexing principles keep evolving over time. For instance, in response to emerging new concepts in the biomedical research, MeSH 2014 includes almost five times more concepts than the edition of MeSH in 1963 that only contains 5,700 descriptors. On the other hand, the articles in PubMed are not re-indexed when MeSH gets updated. Thus, it is not always obvious in selecting benchmarking data sets for system development and comparison.

In this paper, we propose a new method, MeSH Now, to the automatic MeSH indexing task. MeSH Now is built on our previous research [3] but has a number of significant advancements: First, MeSH Now combines different methods through machine learning. Second, new post-processing and list-pruning steps are now added in MeSH Now for improved performance. Third, from a technical perspective, MeSH Now is optimized using the latest MeSH lexicon and recent indexed articles for system training and development. Finally, MeSH Now is implemented to operate in a parallel computing environment, making it possible for large-scale processing needs (e.g., providing computer results of new PubMed articles for assisting human indexing). For evaluation, we first test MeSH Now on a previous dataset that was widely used in benchmarking. Furthermore, we created a new benchmarking dataset based on the recent BioASQ 2014 challenge task data. Our experimental results show that MeSH Now achieves state-of-the-art performance on both data sets.

## Methods

### Approach overview

**Fig. 2:**
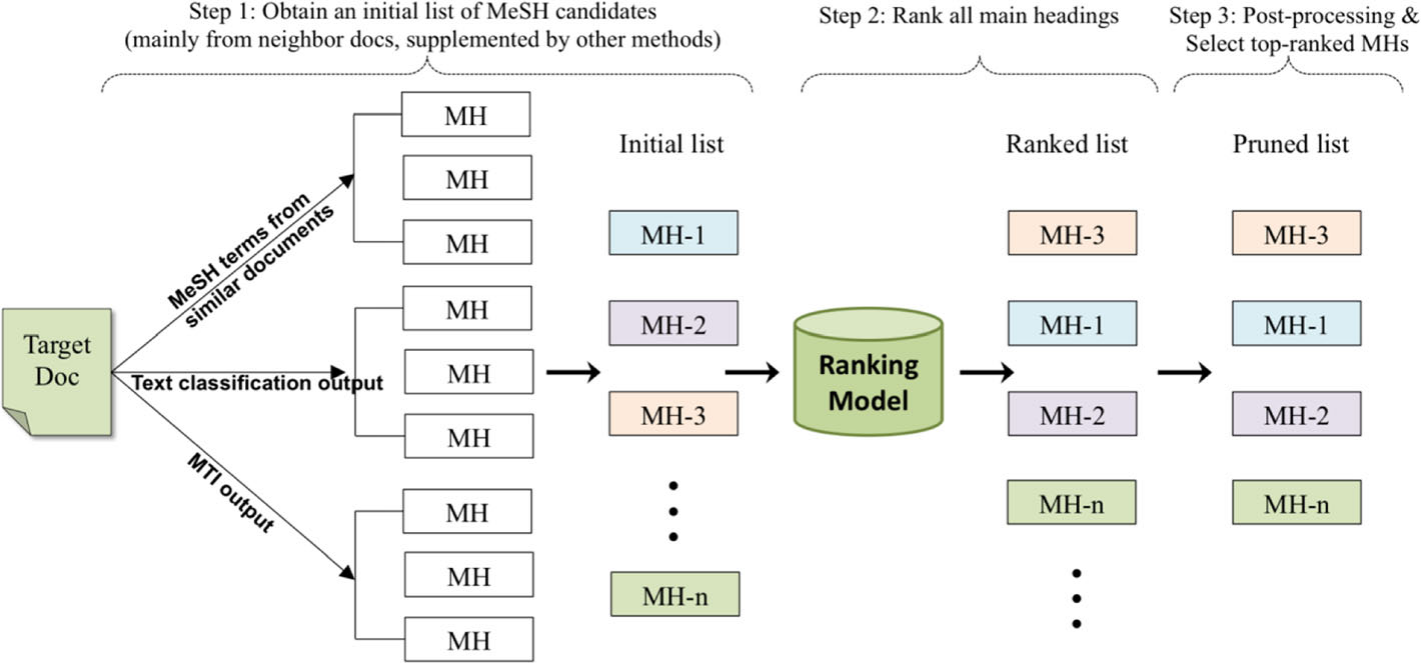
System overview

Our approach reformulates the MeSH indexing task as a ranking problem. Figure 2 shows the three main steps: First, given a target article, we obtain an initial list of candidate MeSH terms from three unique sources. Next, we apply a learning-to-rank algorithm to sort the candidate MeSH terms based on the learned associations between the document text and each candidate MeSH term. Finally, we prune the ranked list and return a number of top candidates as the final system output. Prior to these steps, some standard text processing was performed such as removing stop words and applying a word-stemming algorithm.

### Input source I: K-nearest neighbours

We first adapt the PubMed Related Articles algorithm [56] to retrieve k-nearest neighbours for each new PubMed article. The assumption is that documents similar in content would share similar MeSH term annotations. Previous work [3] has supported this assumption by showing that over 85% of the gold-standard MeSH annotations for a target document are present in its nearest 20 neighbours.

Furthermore, we found that retrieving neighbours from the whole MEDLINE database performed worse than only retrieving neighbours from a subset of the database (e.g., articles in the BioASQ Journal List, or newly published articles). In particular, the results of our approach are best when limiting the neighbour documents to articles indexed in the last 5 years (i.e. the articles were assigned with MeSH terms after 2009). As mentioned before, MeSH terms evolve every year but the articles already indexed will never be re-indexed. The same article would likely be assigned with different MeSH terms in 2014 versus 20 years ago. Thus there are many outdated MeSH terms in those neighbour documents, which can be harmful to the accuracy of our approach. Moreover, the word frequencies are also different in the older and more recent articles, which are closely related to the similarity score for two articles. Therefore, we built our index with only articles that were assigned with MeSH terms after 2009, and retrieved the neighbour documents using such a new index instead of retrieving similar documents from the whole PubMed. When building our document index for the PubMed Related Articles algorithm^3^, we also make sure that all annotated MeSH terms are removed such that they are not used in the computation of the neighbour documents. In other words, the similarity between two documents is solely based on the words they have in common.

The parameter *k* was fixed (*k* = 20) in [3], which means the same number of neighbours will be included for all target articles. However, we observed that some articles may only have a few very similar documents. We therefore adjust the parameter *k* dynamically between 10 to 40 in this work according to the similarity scores of the neighbours: the smaller the average similarity score of the neighbours, the fewer neighbours will be used. Once those *k*-nearest neighbour documents are retrieved, we collect all of the unique MeSH terms associated with those neighbour documents. Note that we only considered the main headings and removed subheadings attached to the main headings.

### Input source #2: multi-label text classification

Motivated by [57], we implemented a multi-label text classification approach where we treat each MeSH concept as a label and build a binary classifier accordingly. More specifically, we first train individual classification models for each of the most frequently indexed 20,000 MeSH terms, as the remaining ones are rarely used in indexing. Then we apply these models to the new article and add those positively classified MeSH concepts as candidates to the initial list. We also keep those associated numerical prediction scores and use them as features in the next step.

Our implementation is based on the cost-sensitive SVM classifiers [58] with Huber loss function [59]. Cost-sensitive SVMs have been shown to be a good solution for dealing with imbalanced and noisy data in biomedical documents [60]. Let *C*_*+*_ denote the higher misclassification cost of the positive class and *C*_*−*_ denote the lower misclassification cost of the negative class, the cost function is formulated as:

$$ \frac{\lambda}{2}{\left\Vert w\right\Vert}^2+{C}_{+}{\displaystyle {\sum}_{i:{y}_i=1} h\left({y}_i\left(\theta + w\cdot {x}_i\right)\right)+{C}_{-}{\displaystyle {\sum}_{i:{y}_i=-1} h\left({y}_i\left(\theta + w\cdot {x}_i\right)\right)}} $$

where MeSH terms are treated as class labels *C* in the classification, *x*_*i*_ is a document of a given class (ie assigned with a specific MeSH term), *λ* is a regularization parameter, *w* is a vector of feature weights, and *θ* is a threshold. The function *h* is the modified Huber loss function and has the form:

$$ h(z)=\left\{\begin{array}{c}\hfill -4\cdot z,\hfill \\ {}\hfill {\left(1- z\right)}^2,\hfill \\ {}\hfill 0,\hfill \end{array}\right.\kern0.48em \begin{array}{c}\hfill z\le -1\hfill \\ {}\hfill -1< z<1\hfill \\ {}\hfill 1\le z\hfill \end{array} $$

We can choose *C*_*+*_ to be greater than *C*_*−*_ to overcome the dominance of negative points in the decision process (here we set *C*_*+*_ = *rC*_*−*_ and the ratio *r* to be 1.5). To train these 20,000 classifiers, we used the MEDLINE articles that were indexed with MeSH terms between January 2009 and March 2014.

### Input source #3: MTI results

MTI is used as one of the baselines in the BioASQ Task, which primarily uses MetaMap to map the phrases in the text to UMLS (Unified Medical Language System) concepts [61]. We thus add all MeSH terms predicted by MTI as candidates, and obtained the feature vectors for those MeSH terms. This is useful since the MTI results can return correct MeSH terms not found by the other two methods.

### Learning to rank

Once an initial list of candidate MeSH terms from all three sources are obtained, we approached the task of MeSH indexing as a ranking problem. In our previous work, we trained the ranking function with ListNet [62], which sorts the results based on a list of scores. In this work we evaluated several other learning-to-rank algorithms [43] on the BioASQ test dataset, including MART [63], RankNet [64], Coordinate Ascent [65], AdaRank [66], and LambdaMART, which are available in RankLib v2.2^4^, and found that LambdaMART achieved the best performance. LambdaMART [67] is a combination of MART and LambdaRank, where the MART algorithm can be viewed as generalizations of logistic regression [63] and LambdaRank is a method for learning arbitrary information retrieval measures [68]. To train such a model, LambdaMART uses gradient boosting to optimize a ranking cost function where the base learners are limited-depth regression trees. New trees are added to an ensemble sequentially that best account for the remaining regression error of the training samples, i.e., each new tree greedily minimizes the cost function. LambdaMART uses MART with specified gradients and Newton’s approximation. LambdaMART is briefly presented as follows [67]:
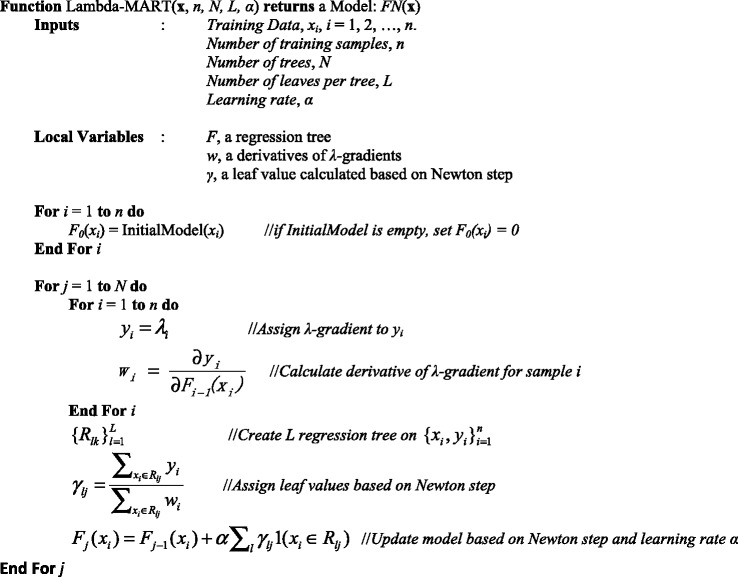


First, we obtained a training set consisting of biomedical articles with human assigned MeSH terms from MEDLINE. For each article, we obtain an initial list of MeSH terms from its neighbour documents. Each MeSH term is then represented as a feature vector. For the list of MeSH terms from its neighbour documents, denoted by {*M*_*1*_ *, M*_*2*_ *, …, M*_*N*_}, where *N* is the number of feature vectors and *M*_*i*_ is the *i*th feature vector, we obtain a corresponding list {*y*_*1*_ *, y*_*2*_ *, …, y*_*N*_}, where *y*_*i*_∈{0,1} is the *i*th class label. *y*_*i*_ = 1 if the MeSH term was manually assigned to the target article by expert indexers of the NLM, otherwise *y*_*i*_ =0.

BioASQ provided approximately 12.6 million PubMed documents for system development. Since all PubMed documents can be used as training data, we randomly selected a set of 5,000 MEDLINE documents from the list of the journals provided by BioASQ for training and optimizing our learning-to-rank algorithm.

### Features

We reused many features developed previously: neighbourhood features, word unigram/bigram overlap features, translation probability features [69], query-likelihood features [70, 71], and synonym features.

For neighbourhood features, we calculate both neighbourhood frequency – the number of times the MeSH term appears in the neighbours, and neighbourhood similarity – the sum of similarity scores for these neighbours.

For translation probability features, we use the IBM translation model [69], which uses title and abstract as source language, and MeSH terms as target language. We then utilize an EM-based algorithm to train the translation probabilities.

For query-likelihood features, we treat each MeSH term as Query (Q), title and abstract as document, and use two genres of query models: classic BM25 model [70] and translation-based query model [71], to calculate the probability of whether a MeSH term should be assigned to the article.

In this work, we added a new domain-specific knowledge feature. We used a binary feature indicating whether a candidate term is observed by MTI, which relies heavily on the domain-specific UMLS Meta-thesaurus [72], for generating its results.

To compute the average length of documents and the document frequency for each word, a set of approximately 60,000 PubMed documents is assembled. These documents are sampled from recent publications in the BioASQ Select Journal List. The translation model and the background language model were built through training with this data set accordingly.

### Post-processing and list pruning

We further improve our results with some post-processing steps.

First, we observed that the Check tags (a special set of MeSH Headings that are mentioned in almost every article such as human, animal, male, female, child, etc.^5^) especially the tags for the age factor are most difficult for our approach. The reason is that the Check tags are frequently present in the neighbour documents, e.g., an article describing a disease in children might have many similar documents discussing about the same disease in adults, which will result in assigning the undesirable Check tag “Adult” to the new article. On the other hand, it is improper to simply exclude the tag “Adult” if “Child” already exists, because many articles in PubMed indeed include both “Adult” and “Child” as MeSH terms. More importantly, many Check tags related to age information are added according to the full text article. In BioASQ, we add the age check tags identified from the abstract text. We first find the numbers near the explicit “age” in the abstract, then predict the correct Age Check Tag according to those numbers and the rules for age check tags.

Second, to improve the precision, we remove the parental MeSH terms when a more specific term is also predicted. This heuristic is based on the principle that indexers should prefer the most specific term applicable instead of more general terms. Therefore in the candidate list, if a child term is ranked higher than its parent term, we will remove the latter accordingly.

Finally, after each MeSH term in the initial list is assigned a score by the ranking algorithm described above, the top *N* ranked MeSH terms will be considered relevant to the target article. *N* was set to be a fixed number (*N* = 25) previously. We found, however, that the average number of MeSH terms per article in the BioASQ training data was only 12.7. Thus, we used an automatic cut-off method to further prune the results from the top ranked MeSH terms as follows:

$$ {S}_{i+1}<{S}_i\cdot \log (i)\cdot \lambda $$

where *S*_*i*_ is the score of the predicted MeSH term at position *i* in the top ranking list. The rationale for Formula (1) is that if the (*i + 1*)th MeSH term was assigned with a score much smaller than the ith MeSH term, the MeSH terms ranked lower than *i* would not be considered relevant to the target article. Formula (1) also accounts for the fact that the difference between lower-ranked MeSH terms is subtler than the difference between higher-ranked MeSH terms. The parameter λ was empirically set to be 0.3 in this research, and it can be tuned to generate predictions favouring either recall or precision.

## Results

### Benchmarking datasets

To demonstrate the progress of our development over time and compare with other systems, we report our system performance on two separate data sets. One of them was widely used in previous studies: NLM2007 [3]. The NLM2007 dataset contains 200 PubMed documents obtained from the NLM indexing initiative^6^. The other is created from the BioASQ 2014 test datasets: BioASQ5000.

In 2014, the BioASQ challenge task [45] ran for six consecutive periods (batches) of 5 weeks each. For each week, the BioASQ organizers distributed new unclassified PubMed documents, and participants have a limited response time (less than 1 day) to return their predicted MeSH terms. As new manual annotations become available, they were used to evaluate the classification performance of participating systems. To be more general (each BioASQ test set contains continuous PMIDs which may belong to a limited set of journals), we randomly selected 5,000 PubMed documents from the latest 9 BioASQ test sets (start from Batch 2 Week 2 in order to avoid overlap with our system training data) to create BioASQ5000, with their corresponding MeSH terms already assigned by December 6, 2014. Compared to NLM2007, BioASQ5000 is much larger in size and contains more recent articles in 2014.

### Comparison of different methods

Here we present our results when evaluated on the two datasets. We first show results on the previously reported benchmarking dataset, NLM2007 [3] in Table 1. For comparison, we show the results of our previous work as “Huang et al., [3]”, and the results of the previous and current versions of MTI (“MTI 2011” and “MTI 2014”). It should be noted that here we used MeSH 2010 and retrieved neighbour documents published before the articles in NLM2007, and our learning-to-rank model was trained with documents published before the articles in NLM2007, because the newly published articles are assigned with new MeSH terms which are not available in NLM2007. We can see that MeSH Now makes significant improvement over our previous method. We also notice that the results of MTI-2014 are much better than those of its previous version. Both MTI-2014 and text classification results (results of input source #2) contribute to the MeSH Now performance with better results generated by MTI than text classification.

**Table 1:** Evaluation results on NLM 2007 test set

Methods	Precision	Recall	F1
MTI – 2011	0.318	0.574	0.409
Huang et al. 2011 [3]	0.390	**0.712**	0.504
Text Classification	**0.655**	0.355	0.461
MTI – 2014	0.568	0.525	0.545
MeSH Now	0.622	0.602	**0.612**

Bold data are the best value

Table 2 shows the results on the BioASQ5000 dataset. For comparison, we added the results of MTI First Line (MTIFL_2014) and MTI Default (MTIDEF_2014), both of which were used as baselines of the BioASQ challenge. This further verifies that our new approach outperforms existing methods.

**Table 2:** Evaluation results on BioASQ5000 test set

Methods	Precision	Recall	F1
Huang et al. 2011 [3]	0.357	**0.701**	0.473
Text Classification	**0.689**	0.400	0.506
MTIFL – 2014	0.621	0.517	0.564
MTI – 2014	0.587	0.559	0.573
MeSH Now	0.612	0.608	**0.610**

Bold data are the best value

### System throughput

The time complexity of large-scale automatic indexing is crucial to real-world systems but rarely discussed in the past. In Table 3, we present the average processing time of each step of our method based on BioASQ5000 on a single computer. We can see that text classification appears to be a bottleneck given the large size of the classifiers (20,000). However, this step can be performed in parallel so that the overall time can be greatly reduced. For example, our current system takes approximately 9 h to process 700,000 articles via a computer cluster where 500 jobs can run concurrently.

**Table 3:** Processing time analysis for different steps

Key steps in MeSH Now	Average time per document (ms)
Obtaining candidate terms via k-NN	1890.82
Obtaining candidate terms via MTI	570.33
Obtaining classification results from each binary text classifier	25.63
Learning to Ranking	103.86
Post-Processing and List Pruning	1.85

## Discussion and conclusions

To better understand the differences between the computer-predicted and human-indexed results, we conducted an error analysis based on the results of MeSH Now on BioASQ5000 dataset. First, we found that the predicted MeSH terms with the lowest performance belong to MeSH Category E: “Analytical, Diagnostic and Therapeutic Techniques and Equipment”, especially the “Statistics as Topic” subcategory, such as “Chi-Square Distribution”, “Survival Analysis”, etc. This is most likely due to the lack of sufficient positive instances in the training set (i.e. the numbers of these indexed terms in the gold standard are relatively small). On the other hand, the most incorrectly predicted MeSH terms are Check Tags (e.g. “Male”, “Female”, “Adult”, “Young Adult”, etc.) despite that the F_1_ scores of these individual Check Tags are reasonably high (most are above the average). Because of their prevalence in the indexing results, however, improving their prediction is critical for increasing the overall performance.

As mentioned before, MeSH Now was developed in 2014 based on the learning-to-rank framework we first proposed in 2010 [3] for automatic MeSH indexing. At the same time, our ranking framework was adopted by several other state-of-the-art systems such as MeSHLabeler [73] and DeepMeSH [74]. MeSHLabeler is very similar to MeSH Now with the major difference in using a machine learning model to predict the number of MeSH terms instead of heuristics. DeepMeSH further incorporates deep semantic representation into MeSHLabeler for improved performance (0.63 in the latest BioASQ challenge in 2016).

There are some limitations and remaining challenges in this work for the automatic MeSH indexing task. First, our previous work revealed that 85% of the gold-standard MeSH annotations should be present in the candidate list based on the nearest 20 neighbours. However, our current best recall is below 65%, suggesting there is still room for improving the learning-to-rank algorithm to promote the relevant MeSH terms higher in the ranked list. Second, our current binary text classification results are lower than previously reported [35], partly because for all classifiers we simply used the same training data, which is quite imbalanced. We believe that the performance of MeSH Now could be further improved if better text classification results are available to be integrated. Finally, we are interested in exploring the opportunities of using MeSH Now in practical applications.

## Footnotes

1
http://www.ncbi.nlm.nih.gov/mesh/

2
http://www.bioasq.org/

3
http://www.ncbi.nlm.nih.gov/books/NBK3827/

4
http://sourceforge.net/p/lemur/wiki/RankLib/

5
http://www.nlm.nih.gov/bsd/indexing/training/CHK_010.html

6
http://ii.nlm.nih.gov/DataSets/

## References

[1] Névéol A, Islamaj Doğan R, Lu Z (2011). Semi-automatic semantic annotation of PubMed queries: a study on quality, efficiency, satisfaction. J Biomed Inform.

[2] Islamaj Dogan R, Murray GC, Neveol A, Lu Z (2009). Understanding PubMed user search behavior through log analysis. Database.

[3] Huang M, Névéol A, Lu Z (2011). Recommending MeSH terms for annotating biomedical articles. J Am Med Inform Assoc.

[4] Lu Z, Kim W, Wilbur WJ (2009). Evaluation of query expansion using MeSH in PubMed. Inf Retr.

[5] Sarkar IN, Schenk R, Miller H, Norton CN. LigerCat: using “MeSH clouds” from journal, article, or gene citations to facilitate the identification of relevant biomedical literature. AMIA Annu Symp Proc. 2009;2009:563–567. PMC2815376 20351918

[6] Smalheiser NR, Zhou W, Torvik VI (2008). Anne O’Tate: A tool to support user-driven summarization, drill-down and browsing of PubMed search results. J Biomed Discov Collab.

[7] Torvik VI, Smalheiser NR (2009). Author name disambiguation in MEDLINE. ACM Trans Knowl Discov Data.

[8] Liu W, Islamaj Doğan R, Kim S, Comeau DC, Kim W, Yeganova L, Lu Z, Wilbur WJ (2014). Author name disambiguation for PubMed. J Assoc Inf Sci Technol.

[9] Bhattacharya S, Ha V, Srinivasan P (2011). MeSH: a window into full text for document summarization. Bioinformatics.

[10] Zhu S, Zeng J, Mamitsuka H (2009). Enhancing MEDLINE document clustering by incorporating MeSH semantic similarity. Bioinformatics.

[11] Jimeno-Yepes AJ, McInnes BT, Aronson AR (2011). Exploiting MeSH indexing in MEDLINE to generate a data set for word sense disambiguation. BMC Bioinformatics.

[12] Perez-Iratxeta C, Andrade-Navarro MA, Wren JD (2007). Evolving research trends in bioinformatics. Brief Bioinform.

[13] DeShazo JP, LaVallie DL, Wolf FM (2009). Publication trends in the medical informatics literature: 20 years of. BMC Med Inform Decis Mak.

[14] D’Souza JL, Smalheiser NR (2014). Three journal similarity metrics and their application to biomedical journals. PLoS One.

[15] Boyack KW (2004). Mapping knowledge domains: Characterizing PNAS. Proc Natl Acad Sci.

[16] Burrows SC, Tylman V (1999). Evaluating medical student searches of MEDLINE for evidence-based information: process and application of results. Bull Med Libr Assoc.

[17] Gruppen LD, Rana GK, Arndt TS (2005). A controlled comparison study of the efficacy of training medical students in evidence-based medicine literature searching skills. Acad Med.

[18] Tennant MR, Miyamoto MM (2002). The role of medical libraries in undergraduate education: a case study in genetics. J Med Libr Assoc.

[19] Jani SD, Argraves GL, Barth JL, Argraves WS (2010). GeneMesh: a web-based microarray analysis tool for relating differentially expressed genes to MeSH terms. BMC Bioinformatics.

[20] Masys DR, Welsh JB, Fink JL, Gribskov M, Klacansky I, Corbeil J (2001). Use of keyword hierarchies to interpret gene expression patterns. Bioinformatics.

[21] Mottaz A, Yip YL, Ruch P, Veuthey A-L (2008). Mapping proteins to disease terminologies: from UniProt to MeSH. BMC Bioinformatics.

[22] Sartor MA, Ade A, Wright Z, Omenn GS, Athey B, Karnovsky A (2012). Metab2MeSH: annotating compounds with medical subject headings. Bioinformatics.

[23] Cheung WA, Ouellette BF, Wasserman WW (2012). Inferring novel gene-disease associations using medical subject heading over-representation profiles. Genome Med.

[24] Ono T, Kuhara S (2014). A novel method for gathering and prioritizing disease candidate genes based on construction of a set of disease-related MeSH (R) terms. BMC Bioinformatics.

[25] Nakazato T, Takinaka T, Mizuguchi H, Matsuda H, Bono H, Asogawa M (2008). BioCompass: a novel functional inference tool that utilizes MeSH hierarchy to analyze groups of genes. In Silico Biol.

[26] Khare R, Li J, Lu Z. LabeledIn: cataloging labeled indications for human drugs. J Biomed Inform. 2014;52:448–456. 10.1016/j.jbi.2014.08.004 PMC4260997 25220766

[27] Lu Z, Hirschman L (2012). Biocuration workflows and text mining: overview of the BioCreative 2012 Workshop Track II. Database.

[28] Mao Y, Van Auken K, Li D, Arighi CN, McQuilton P, Hayman GT, Tweedie S, Schaeffer ML, Laulederkind SJ, Wang S-J (2014). Overview of the gene ontology task at BioCreative IV. Database.

[29] Van Auken K, Schaeffer ML, McQuilton P, Laulederkind SJ, Li D, Wang S-J, Hayman GT, Tweedie S, Arighi CN, Done J (2014). BC4GO: a full-text corpus for the BioCreative IV GO task. Database.

[30] Lu Z, Cohen KB, Hunter L. GeneRIF quality assurance as summary revision. Pac Symp Biocomput. 2007:269–280. 10.1142/9789812772435_0026 PMC2652871 17990498

[31] Huang M, Lu Z. Learning to annotate scientific publications. In: Proceedings of the 23rd International Conference on Computational Linguistics: Posters. Stroudsburg: Association for Computational Linguistics; 2010. pp. 463–471.

[32] Aronson AR. Effective mapping of biomedical text to the UMLS Metathesaurus: the MetaMap program. In: Proceedings of the AMIA Symposium. Washington DC; 2001. pp. 17–21. PMC2243666 11825149

[33] Ruiz ME, Srinivasan P (2002). Hierarchical text categorization using neural networks. Inf Retr.

[34] Yetisgen-Yildiz M, Pratt W. The effect of feature representation on MEDLINE document classification. In: AMIA annual symposium proceedings. Washington D.C: American Medical Informatics Association; 2005. pp. 849–853. PMC1560754 16779160

[35] Tsoumakas G, Laliotis M, Markantonatos N, Vlahavas IP (2013). Large-scale semantic indexing of biomedical publications. BioASQ@ CLEF.

[36] Névéol A, Shooshan SE, Claveau V (2008). Automatic inference of indexing rules for MEDLINE. BMC Bioinformatics.

[37] Sohn S, Kim W, Comeau DC, Wilbur WJ (2008). Optimal training sets for bayesian prediction of MeSH® assignment. J Am Med Inform Assoc.

[38] Wilbur WJWK (2014). Stochastic gradient descent and the prediction of MeSH for PubMed records. AMIA.

[39] Jimeno-Yepes A, Mork JG, Demner-Fushman D, Aronson AR (2012). A one-size-fits-all indexing method does not exist: automatic selection based on meta-learning. JCSE.

[40] Yang Y, Chute CG. An application of Expert Network to clinical classification and MEDLINE indexing. The 18th Annual Symposium on Computer Applications in Medical Care. Bethesda: American Medical Informatics Association; 1994. pp. 157–161. PMC2247915 7949911

[41] Trieschnigg D, Pezik P, Lee V, De Jong F, Kraaij W, Rebholz-Schuhmann D (2009). MeSH Up: effective MeSH text classification for improved document retrieval. Bioinformatics.

[42] Delbecque T, Zweigenbaum P. Using Co-Authoring and Cross-Referencing Information for MEDLINE Indexing. In: AMIA Annual Symposium Proceedings. Washington DC: American Medical Informatics Association; 2010. pp. 147–151. PMC3041281 21346958

[43] Liu T-Y (2009). Learning to rank for information retrieval. Found Trends Inf Retr.

[44] Mao Y, Wei C-H, Lu Z (2014). NCBI at the 2014 BioASQ challenge task: large-scale biomedical semantic indexing and question answering. Proceedings of Question Answering Lab at CLEF.

[45] Balikas G, Partalas I, Ngomo A-CN, Krithara A, Gaussier E, Paliouras G. Results of the BioASQ Track of the Question Answering Lab at CLEF 2014. In: Proceedings of Question Answering Lab at CLEF. 2014. pp. 1181–1193.

[46] Tsatsaronis G, Balikas G, Malakasiotis P, Partalas I, Zschunke M, Alvers MR, Weissenborn D, Krithara A, Petridis S, Polychronopoulos D (2015). An overview of the BIOASQ large-scale biomedical semantic indexing and question answering competition. BMC Bioinformatics.

[47] Liu K, Wu J, Peng S, Zhai C, Zhu S (2014). The Fudan-UIUC participation in the BioASQ Challenge Task 2a: The Antinomyra system. Risk.

[48] Kavuluru R, Lu Y. Leveraging output term co-occurrence frequencies and latent associations in predicting medical subject headings. Data & Knowledge Engineering. 2014;94:189–201. 10.1016/j.datak.2014.09.002 PMC5524140 28747808

[49] Mork JG, Jimeno-Yepes A, Aronson AR (2013). The NLM Medical Text Indexer System for Indexing Biomedical Literature. BioASQ@ CLEF.

[50] Ruch P (2006). Automatic assignment of biomedical categories: toward a generic approach. Bioinformatics.

[51] Aronson AR, Mork JG, Gay CW, Humphrey SM, Rogers WJ (2004). The NLM indexing initiative’s medical text indexer. Medinfo.

[52] Névéol A, Shooshan SE, Humphrey SM, Mork JG, Aronson AR (2009). A recent advance in the automatic indexing of the biomedical literature. J Biomed Inform.

[53] Mork JG, Demner-Fushman D, Schmidt SC, Aronson AR (2014). Recent enhancements to the NLM medical text indexer. Working Notes for CLEF 2014 Conference, Sheffield, UK.

[54] Partalas I, Gaussier É, Ngomo A-CN (2013). Results of the First BioASQ Workshop. BioASQ@ CLEF.

[55] Funk ME, Reid CA (1983). Indexing consistency in MEDLINE. Bull Med Libr Assoc.

[56] Lin J, Wilbur WJ (2007). PubMed related articles: a probabilistic topic-based model for content similarity. BMC Bioinformatics.

[57] Tang L, Rajan S, Narayanan VK. Large scale multi-label classification via metalabeler. In: Proceedings of the 18th international conference on World wide web. New York: ACM; 2009. pp. 211–220.

[58] Thai-Nghe N, Gantner Z, Schmidt-Thieme L. Cost-sensitive learning methods for imbalanced data. In: Proceedings of the IEEE International Joint Conference on Neural Networks (IJCNN 2010), Barcelona, Spain. 2010. pp. 1–8.

[59] Huber PJ (1964). Robust estimation of a location parameter. Ann Math Stat.

[60] Kim W, Yeganova L, Comeau DC, Wilbur WJ. Identifying well-formed biomedical phrases in MEDLINE® text. J Biomed Inform. 2012;45(6):1035–1041. 10.1016/j.jbi.2012.05.005 PMC3465642 22683889

[61] Yepes AJJ, Mork JG, Demner-Fushman D, Aronson AR. Comparison and combination of several MeSH indexing approaches. In: AMIA Annual Symposium Proceedings. Washington DC: American Medical Informatics Association; 2013. pp. 709–718. PMC3900212 24551371

[62] Cao Z, Qin T, Liu T-Y, Tsai M-F, Li H. Learning to rank: from pairwise approach to listwise approach. In: Proceedings of the 24th international conference on Machine learning. New York: ACM; 2007. pp 129–136.

[63] Friedman JH (2001). Greedy function approximation: a gradient boosting machine. Ann Stat..

[64] Burges C, Shaked T, Renshaw E, Lazier A, Deeds M, Hamilton N, Hullender G. Learning to rank using gradient descent. In: Proceedings of the 22nd international conference on Machine learning. New York: ACM; 2005. pp. 89–96.

[65] Metzler D, Croft WB (2007). Linear feature-based models for information retrieval. Inf Retr.

[66] Xu J, Li H. Adarank: a boosting algorithm for information retrieval. In: Proceedings of the 30th annual international ACM SIGIR conference on Research and development in information retrieval. New York: ACM; 2007. pp. 391–398.

[67] Wu Q, Burges CJ, Svore KM, Gao J (2010). Adapting boosting for information retrieval measures. Inf Retr.

[68] Quoc C, Le V (2007). Learning to rank with nonsmooth cost functions. NIPS’07.

[69] Brown PF, Pietra VJD, Pietra SAD, Mercer RL (1993). The mathematics of statistical machine translation: Parameter estimation. Comput Linguist.

[70] Robertson SE, Walker S, Jones S, Hancock-Beaulieu MM, Gatford M. Okapi at TREC-3. Gaithersburg: NIST Special Publication; 1995. pp. 109–126

[71] Berger A, Lafferty J. Information retrieval as statistical translation. In: Proceedings of the 22nd annual international ACM SIGIR conference on Research and development in information retrieval. New York: ACM; 1999. pp. 222–229.

[72] Humphreys BL, Lindberg DA (1993). The UMLS project: making the conceptual connection between users and the information they need. Bull Med Libr Assoc.

[73] Liu K, Peng S, Wu J, Zhai C, Mamitsuka H, Zhu S (2015). MeSHLabeler: improving the accuracy of large-scale MeSH indexing by integrating diverse evidence. Bioinformatics.

[74] Peng S, You R, Wang H, Zhai C, Mamitsuka H, Zhu S (2016). DeepMeSH: deep semantic representation for improving large-scale MeSH indexing. Bioinformatics.

